# Background parenchymal uptake on molecular breast imaging as a breast cancer risk factor: a case-control study

**DOI:** 10.1186/s13058-016-0704-6

**Published:** 2016-04-26

**Authors:** Carrie B. Hruska, Christopher G. Scott, Amy Lynn Conners, Dana H. Whaley, Deborah J. Rhodes, Rickey E. Carter, Michael K. O’Connor, Katie N. Hunt, Kathleen R. Brandt, Celine M. Vachon

**Affiliations:** Department of Radiology, Mayo Clinic, 200 First Street SW, Rochester, MN 55905 USA; Department of Health Sciences Research, Mayo Clinic, 200 First Street SW, Rochester, MN 55905 USA; Department of Medicine, Mayo Clinic, 200 First Street SW, Rochester, MN 55905 USA

**Keywords:** Breast density, Breast cancer risk, Molecular breast imaging, Tc-99m sestamibi, Mammography

## Abstract

**Background:**

Molecular breast imaging (MBI) is a functional test used for supplemental screening of women with mammographically dense breasts. Additionally, MBI depicts variable levels of background parenchymal uptake (BPU) within nonmalignant, dense fibroglandular tissue. We investigated whether BPU is a risk factor for breast cancer.

**Methods:**

We conducted a retrospective case-control study of 3027 eligible women who had undergone MBI between February 2004 and February 2014. Sixty-two incident breast cancer cases were identified. A total of 179 controls were matched on age, menopausal status, and MBI year. Two radiologists blinded to case status independently assessed BPU as one of four categories: photopenic, minimal to mild, moderate, or marked. Conditional logistic regression analysis was performed to estimate the associations (OR) of BPU categories (moderate or marked vs. minimal to mild or photopenic) and breast cancer risk, adjusted for other risk factors.

**Results:**

The median age was 60.2 years (range 38–86 years) for cases vs. 60.2 years (range 38–88 years) for controls (*p* = 0.88). Women with moderate or marked BPU had a 3.4-fold (95 % CI 1.6–7.3) and 4.8-fold (95 % CI 2.1–10.8) increased risk of breast cancer, respectively, compared with women with photopenic or minimal to mild BPU, for two radiologists. The results were similar after adjustment for BI-RADS density (OR 3.3 [95 % CI 1.6–7.2] and OR 4.6 [95 % CI 2.1–10.5]) or postmenopausal hormone use (OR 3.6 [95 % CI 1.7–7.7] and OR 5.0 [95 % CI 2.2–11.4]). The association of BPU with breast cancer remained in analyses limited to postmenopausal women only (OR 3.8 [95 % CI 1.5–9.3] and OR 4.1 [95 % CI 1.6–10.2]) and invasive breast cancer cases only (OR 3.6 [95 % CI 1.5–8.8] and OR 4.4 [95 % CI 1.7–11.1]). Variable BPU was observed among women with similar mammographic density; the distribution of BPU categories differed across density categories (*p* < 0.0001).

**Conclusions:**

This study provides the first evidence for BPU as a risk factor for breast cancer. Among women with dense breasts, who comprise >40 % of the screening population, BPU may serve as a functional imaging biomarker to identify the subset at greatest risk.

**Electronic supplementary material:**

The online version of this article (doi:10.1186/s13058-016-0704-6) contains supplementary material, which is available to authorized users.

## Background

Mammographic density is known to be an important factor in reducing the sensitivity of mammography [1, 2]. Therefore, supplemental screening options capable of detecting mammographically occult cancers are now increasingly being offered to women with dense breasts [3–5]. Density is also strongly associated with breast cancer risk, even after accounting for its masking of cancers [1, 6]. However, density alone lacks sufficient discriminatory accuracy to be clinically useful in individual risk assessment [6, 7]. Risk models developed within the Breast Cancer Surveillance Consortium (BCSC) have incorporated American College of Radiology (ACR) Breast Imaging-Reporting and Data System (BI-RADS) breast density categories [8], a genetic risk score, and history of benign breast disease; yet, c-statistics, or AUC, remain <0.67 [9, 10]. Given the high prevalence of dense breasts (43 % of screening-eligible U.S. women have heterogeneously or extremely dense breasts according to BI-RADS criteria [11]), coupled with increasing adoption of breast density notification laws (in 26 states to date [12]), further tools are needed to identify women most likely to benefit from supplemental screening or primary prevention.

Molecular breast imaging (MBI) is a functional imaging technique that uses a specialized gamma camera to detect preferential uptake of Tc-99m sestamibi in metabolically active breast tissue. When used as a supplement to screening mammography in women with dense breasts, MBI showed an incremental cancer detection rate of 8.8 per 1000 women screened [5]. MBI can also show variable levels of radiotracer uptake within areas of nonmalignant fibroglandular tissue, a finding termed *background parenchymal uptake* (BPU) [13]. BPU is associated with hormonal influences, including menopausal status, postmenopausal hormone use, and phase of menstrual cycle [14, 15]. Also, BPU varies among women with similar mammographic density [14].

We hypothesized that the functional differences reflected in MBI background provide additional information for determining breast cancer risk in women with dense breasts. Here, our objective was to investigate whether BPU on MBI is a risk factor for breast cancer.

## Methods

### Study population

We performed a retrospective case-control study that was compliant with the U.S. Health Insurance Portability and Accountability Act and approved by the Mayo Clinic Institutional Review Board, which issued a waiver of informed consent. Our institutional MBI database was reviewed to identify all patients (*n* = 3202) who had had at least one MBI examination performed at the Mayo Clinic in Rochester, MN, USA, between 1 February 2005 and 28 February 2014. The Mayo Clinic patients provides a general authorization for use of medical record information for research purposes. We included only women who provided this authorization (*n* = 3085 [96 %] of 3202). For women who had had multiple MBI examinations performed, data from the earliest (index) MBI were used for analysis. Follow-up for breast cancer through December 2014 was conducted through review of medical records and linkage to the Mayo Clinic Tumor Registry.

Women with breast implants at the time of index MBI were excluded, as photopenia due to the presence of implants makes assessment of BPU difficult. Women with a history of breast cancer before the index MBI examination or a diagnosis within 180 days following the index MBI were also excluded to minimize prevalent cases, as done in prior studies [16]. After these exclusions, 3027 eligible participants remained from among whom to identify cases and controls. Average follow-up time of participants was 4.6 years.

Incident cases were defined as participants with a histopathologic diagnosis of ductal carcinoma in situ (DCIS) or invasive carcinoma in either breast at least 180 days after the index MBI. Sixty-two incident breast cancer cases were identified. The median time between index MBI examination and cancer diagnosis was 3.1 years (IQR 1.5–4.2 years). Fifty-eight (94 %) of sixty-two cases were diagnosed more than 1 year after the index MBI.

Up to three control subjects per case (*n* = 179) were selected from among the 2965 women who were not diagnosed with breast cancer over the study period, with matching to cases on age (within 5 years), menopausal status (exact), and year of MBI (within 1 year). Matched controls were required to be followed for at least as long as matched cases and to have had negative findings on all subsequent breast imaging performed at our institution over the corresponding follow-up time. Median follow-up time for controls was 6.1 years (IQR 3.7–7.8 years).

Of the 241 women studied, MBI was performed as a supplemental screen to mammography in 228 (95 %, comprising 57 cases and 171 controls). In the remaining 13 women (5 %), MBI was performed for further evaluation of the following: breast mass initially detected on mammography or ultrasound (three cases, five controls), recent histopathologic diagnosis of atypia or lobular carcinoma in situ (one case, two controls), bloody nipple discharge (one case), and breast pain (one control).

Covariate information, including body mass index (BMI), menopausal status, postmenopausal hormone use, breast biopsy history, and family history of breast cancer was obtained primarily in a prospective manner through questionnaires and medical record review performed at the time of the MBI examination. We examined only those factors previously shown to be associated with BPU [14]. Missing information was retrospectively abstracted by research nurses.

### Molecular breast imaging procedure

MBI examinations were performed using one of two dedicated dual-head gamma camera systems equipped with cadmium zinc telluride detectors (LumaGEM, Gamma Medica, Salem, NH, USA; or Discovery NM750b, GE Healthcare, Haifa, Israel). Patients received an intravenous injection of Tc-99m sestamibi in an arm vein. Before June 2009, MBI examinations were performed with administered doses of 740 MBq of Tc-99 m sestamibi. Changes to the MBI detectors’ collimation, system energy window settings, and radiopharmaceutical injection techniques over the course of the study period resulted in an approximately threefold gain in the number of counts that could be collected during an MBI examination, such that we were able to lower the administered dose to patients proportionally while preserving count density and image quality [17, 18]. In June 2009 and later, the administered dose was between 240 and 300 MBq of Tc-99m sestamibi. Matching of cases and controls by year of MBI served to control for any changes in MBI examinations before and after the protocol change.

Imaging commenced approximately 5 minutes after injection. Bilateral views in craniocaudal and mediolateral oblique-analogous projections were acquired for 10 minutes per view, with the breast under light compression to limit patient motion. All study participants had bilateral MBI examinations.

### Background parenchymal uptake assessment

**Fig. 1:**
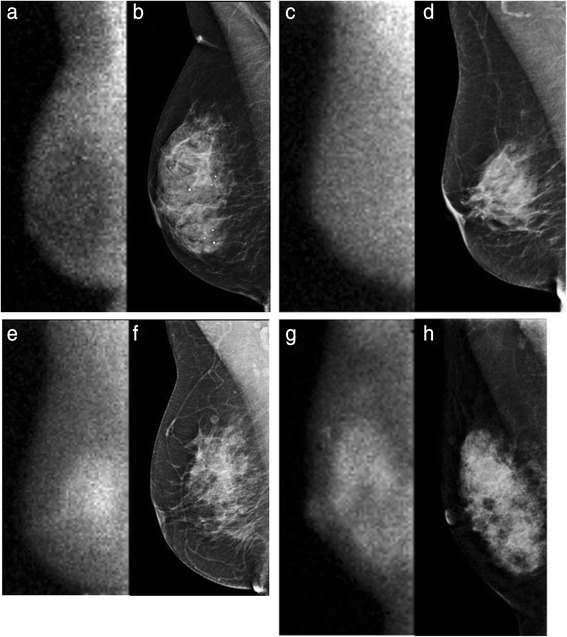
Examples of background parenchymal uptake categories. Molecular breast imaging (MBI) examinations and corresponding full-field digital mammograms from four different women are shown. All images were acquired in the mediolateral oblique projection. MBI with photopenic background parenchymal uptake (BPU) (**a**) and corresponding mammogram (**b**). MBI with minimal to mild BPU (**c**) and corresponding mammogram (**d**). MBI with moderate BPU (**e**) and corresponding mammogram (**f**). MBI with marked BPU (**g**) and corresponding mammogram (**h**)

MBI examinations were retrospectively reviewed by two breast imaging radiologists with 6 and 10 years of experience, respectively, in MBI interpretation. Readers interpreted BPU independently while blinded to the participants’ case status. BPU was qualitatively assessed per breast according to a validated lexicon for gamma imaging of the breast as one of four categories: photopenic, minimal to mild, moderate, or marked (Fig. 1) [19]. These categories describe the relative intensity of radiotracer uptake observed in areas of normal parenchyma compared with that in areas of subcutaneous fat, as follows: photopenic BPU, parenchymal intensity less than fat intensity; minimal to mild BPU, parenchymal intensity equal to or slightly greater than fat intensity; moderate BPU, parenchymal intensity greater than mild but less than twice as intense as fat; and marked, parenchymal intensity greater than twice fat intensity.

### Mammographic density assessment

Mammographic density was assessed on mammograms obtained closest to the time of MBI. Mammograms were available for 232 (96 %) of 241 subjects. Most mammograms were obtained within 21 days of MBI (223 [96 %] of 232). Six were obtained within 6 months, and the remaining three were obtained within 14 months of MBI. During the course of routine clinical practice, mammographic density was subjectively assessed according to ACR BI-RADS density categories (fourth edition) by breast imaging radiologists [8]. Density was also quantitatively assessed as percentage density by a trained operator using semiautomated software (Cumulus; University of Toronto, Toronto, ON, Canada [20]) as previously described [14, 21]. Percentage density was measured on right and left craniocaudal mammograms from either digitized film or “for presentation” digital mammograms in Digital Imaging and Communications in Medicine format.

### Statistical analysis

Case and control characteristics are presented as mean, SD, and range for continuous variables and frequency and percentage for categorical variables. Conditional logistic regression analysis was performed to test for differences between cases and controls and to estimate the associations of BPU categories and risk of breast cancer with adjustments for covariates. Exploratory analyses were also repeated within premenopausal and postmenopausal subgroups and in a subgroup limited to invasive breast cancer cases. Further, we performed analyses on data from before and after June 2009 to examine whether changes in MBI protocol influenced the results. To measure the discriminatory ability of the models, the AUC for BPU discrimination was calculated within case-control sets to match the design.

Analysis was performed by first considering BPU as a four-category variable, using the category “minimal to mild” as a reference group because this was the largest group and provided a more stable relative risk estimate. Tests for trend were examined by including BPU as an ordinal variable in the conditional logistic regression model. Analysis was also performed considering BPU as a dichotomous variable by combining categories of “photopenic” with “minimal to mild” and “moderate” with “marked.”

The maximum BPU was used in participants whose BPU assessment differed between breasts, similarly to how clinical assessment of BI-RADS density is done [8]. To assess whether the associations were different for BPU assessed on contralateral vs. ipsilateral breasts, analyses were also done stratified by side as defined by cancer location. For this analysis, the side of the matched case was used to define the side for controls.

Interreader agreement in BPU assessment was summarized by the weighted κ statistic. κ values were interpreted as ≤0.20 or less, poor; 0.21–0.40, fair; 0.41–0.60, moderate; 0.61–0.80, good; and 0.81–1.00, very good agreement [22].

The χ^2^ test was used as appropriate to test for differences in distribution of BPU categories across BI-RADS density categories. For all comparisons, *p* < 0.05 was considered statistically significant. Analyses were performed using SAS software version 9.4 (SAS Institute, Cary, NC, USA).

## Results

### Study group characteristics

Characteristics of breast cancer cases and matched controls are shown in Table 1. As expected, cases and controls were similar on matched variables of age and menopausal status. Nearly all subjects (174 [97 %] of 179) were age-matched exactly; the remaining five subjects were age-matched within 1–3 years. Breast cancer cases had higher percentages of family history, history of atypia or lobular carcinoma in situ, mean BMI, Gail model 5-year risk, and BCSC model 5-year risk, but none of these reached statistical significance.

**Table 1:** Characteristics of breast cancer cases and controls, matched on age, menopausal status, and MBI year

Characteristic	Breast cancer cases (*n* = 62)	Controls (*n* = 179)	*p* value
Age at MBI,^a^years	60.3 ± 10.6 (38–86)	60.2 ± 10.6 (38–88)	0.88
Menopausal status			N/A^b^
Premenopausal	13 (21)	38 (21)	
Postmenopausal	49 (79)	141 (79)	
BMI^a^	27.7 ± 6.4 (18.8–55.5)	26.2 ± 4.6 (18.6–44.3)	0.08
Postmenopausal systemic HRT^c^			0.57
Current use at MBI	13 (27)	44 (31)	
No current use at MBI	36 (73)	97 (69)	
BI-RADS density			0.77
Almost entirely fat	1 (2)	3 (2)	
Scattered fibroglandular densities	10 (16)	34 (19)	
Heterogeneously dense	44 (71)	114 (64)	
Extremely dense	7 (11)	26 (15)	
Unknown	0	2 (1)	
Percentage density^a^	24.8 ± 8.3 (3.5–48.0)	24.6 ± 10.2 (1.8–53.8)	0.92
MBI protocol			0.88
740 MBq Tc-99m sestamibi	36 (58)	102 (57)	
240–300 MBq of Tc-99m sestamibi	26 (42)	77 (43)	
Tumor invasiveness			
Invasive	45 (73)	NA	
DCIS	17 (27)	NA	
Gail model 5-year risk^a^	2.7 ± 1.5 (0.6–7.2)	2.4 ± 1.5 (0.5–9.5)	0.23
BCSC model 5-year risk^a^	2.6 ± 1.2 (0.7–5.4)	2.3 ± 1.5 (0.4–13.2)	0.29
Family history of breast cancer			0.45
One or more first-degree relatives	33 (53)	86 (48)	
No first-degree relatives	29 (47)	93 (52)	
Personal history of biopsy showing atypia or LCIS			0.07
Yes	6 (10)	6 (3)	
No	56 (90)	173 (97)	

*BCSC* Breast Cancer Surveillance Consortium, *BI-RADS* Breast Imaging-Reporting and Data System, *BMI* body mass index, *DCIS* ductal carcinoma in situ, *HRT* hormone replacement therapy, *LCIS* lobular carcinoma in situ, *MBI* molecular breast imaging

*Note*: Unless otherwise noted, data are number of patients and data in parentheses are percentages

^a^Data are mean ± SD. Data in parentheses are ranges.

^b^Exact matches

^c^Data are among postmenopausal women only (49 breast cancer cases, 141 controls)

**Fig. 2:**
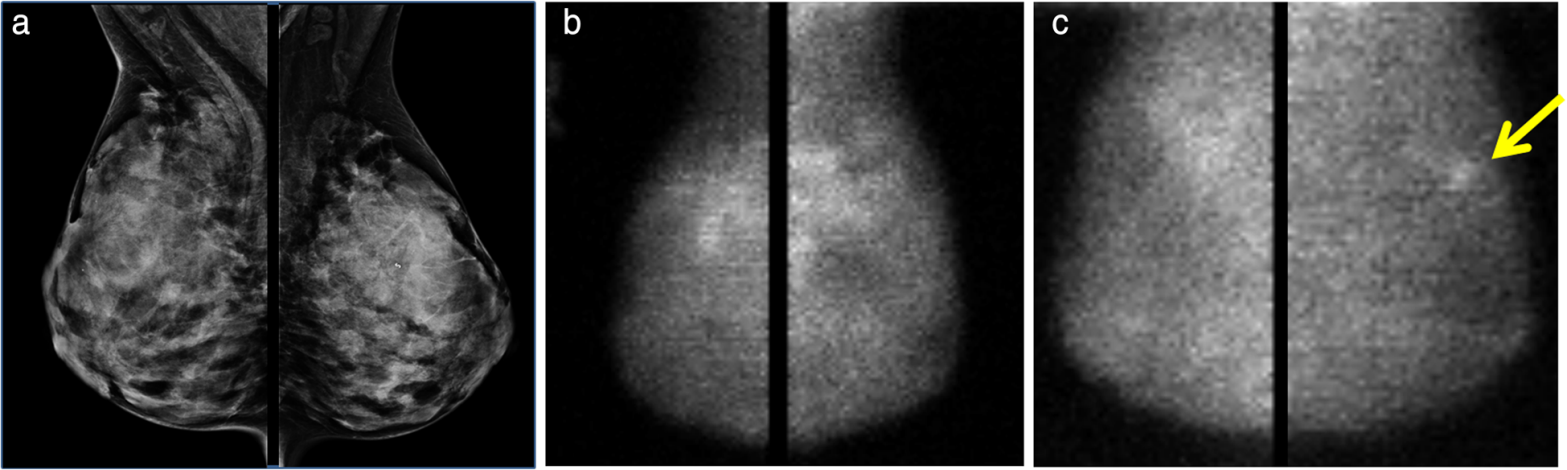
Example of incident breast cancer case. Right and left mediolateral oblique views are shown. In this 41-year-old premenopausal woman, screening full-field digital mammography (**a**) was negative with Breast Imaging-Reporting and Data System density category of extremely dense. Supplemental screening molecular breast imaging (MBI) (**b**) performed at the time of the screening mammogram showed marked background parenchymal uptake. The next 2 years of screening mammography were negative. At 2.6 years after the initial MBI, the patient presented for diagnostic workup of nipple retraction. (**c**) MBI performed at the time showed a lesion in the left breast (*arrow*) that was diagnosed as a 9-mm, grade I invasive ductal carcinoma

Of the 62 incident breast cancer cases, 45 (73 %) were invasive and 17 (27 %) were DCIS. Images from an example breast cancer case are given in Fig. 2. Details of cases are given in Additional file 1: Table S1.

### BPU agreement

When BPU was considered as a four-category ordinal variable, interreader agreement was good (κ = 0.77, 95 % CI 0.70–0.84) with 85 % (205 of 241) agreement. When BPU was considered as a dichotomous variable, interreader agreement was good to very good (κ = 0.82, 95 % CI 0.74–0.92) with 94 % (227 of 241) agreement. BPU differed between breasts in 10 (4 %) of 241 participants (5 cases, 5 controls) for reader 1 and in 24 (10 %) of 241 participants (10 cases, 14 controls) for reader 2.

### Breast cancer cases vs. controls

BPU level using either categorization was associated with breast cancer risk. When BPU was considered as a four-category variable, the BMI-adjusted OR increased with increasing BPU (Table 2). For reader 1, the OR increased from 0.8 for photopenic BPU to 3.0 for marked BPU (*p*-trend = 0.007). For reader 2, the OR increased from 0.5 for photopenic BPU to 6.2 for marked BPU (*p*-trend < 0.001).

**Table 2:** Association of four-category background parenchymal uptake at molecular breast imaging with breast cancer

BPU level	Breast cancer cases^a^ (*n* = 62)	Controls^a^ (*n* = 179)	OR^b^	*p*-trend	AUC^b^
Reader 1				0.007	0.62 (0.55–0.69)
Photopenic	5 (8)	26 (15)	0.8 (0.3–2.3)		
Minimal to mild	35 (56)	124 (69)	1.0		
Moderate	12 (19)	12 (7)	3.6 (1.4–8.9)		
Marked	10 (16)	17 (9)	3.0 (1.1–8.4)		
Reader 2				<0.001	0.63 (0.56–0.70)
Photopenic	2 (3)	18 (10)	0.5 (0.1–2.3)		
Minimal to mild	36 (58)	132 (74)	1.0		
Moderate	13 (21)	17 (9)	3.9 (1.6–9.7)		
Marked	11 (18)	12 (7)	6.2 (2.0–19.0)		

*BPU* background parenchymal uptake

^a^Numbers in parentheses are percentages

^b^Numbers in parentheses are 95 % CIs. OR is adjusted for body mass index

When we considered BPU as a dichotomous variable, we found that women with moderate or marked BPU had a greater risk of incident breast cancer than women with photopenic or minimal to mild BPU. For the two readers, the BMI-adjusted ORs for moderate or marked BPU vs. photopenic or minimal to mild BPU was 3.4 (95 % CI 1.6–7.3) and 4.8 (95 % CI 2.1–10.8) (Table 3). When the dataset was limited to invasive breast cancer cases and respective matched controls, the results were similar: BMI-adjusted ORs were 3.6 (95 % CI 1.5–8.8) and 4.4 (95 % CI 1.7–11.1) for the two readers.

**Table 3:** Association of background parenchymal uptake with breast cancer by menopausal status

BPU	Breast cancer cases^a^	Controls^a^	OR adjusted for BMI^b^	OR adjusted for BMI and BI-RADS density^b^	OR adjusted for BMI and postmenopausal HRT^b^
Overall					
Reader 1					
Photopenic or minimal to mild	40/62 (65)	150/179 (84)	1.0	1.0	1.0
Moderate or marked	22/62 (35)	29/179 (16)	3.4 (1.6–7.3)	3.3 (1.6–7.2)	3.6 (1.7–7.7)
*p* value			0.002	0.002	0.001
AUC			0.63 (0.56–0.70)	0.64 (0.57–0.71)	0.63 (0.56–0.70)
Reader 2					
Photopenic or minimal to mild	38/62 (61)	150/179 (84)	1.0	1.0	1.0
Moderate or marked	24/62 (39)	29/179 (16)	4.8 (2.1–10.8)	4.6 (2.1–10.5)	5.0 (2.2–11.4)
*p* value			<0.001	<0.001	<0.001
AUC			0.65 (0.58–0.72)	0.67 (0.60–0.74)	0.65 (0.58–0.72)
Premenopausal women					
Reader 1					
Photopenic or minimal to mild	4/13 (31)	21/38 (55)	1.0	1.0	NA
Moderate or marked	9/13 (69)	17/38 (45)	2.5 (0.6–10.0)	3.0 (0.7–6.8)	
*p* value			0.18	0.14	
AUC			0.63 (0.48–0.78)	0.63 (0.48–0.78)	
Reader 2					
Photopenic or minimal to mild	2/13 (15)	21/38 (55)	1.0	1.0	NA
Moderate or marked	11/13 (85)	17/38 (45)	9.4 (1.1–79.5)	10.2 (1.2–90.4)	
*p* value			0.04	0.04	
AUC			0.68 (0.54–0.83)	0.74 (0.60–0.87)	
Postmenopausal women					
Reader 1					
Photopenic or minimal to mild	36/49 (73)	129/141 (91)	1.0	1.0	1.0
Moderate or marked	13/49 (27)	12/141 (9)	3.8 (1.5–9.3)	3.6 (1.5–9.0)	4.1 (1.6–10.3)
*p* value			0.004	0.006	0.003
AUC			0.64 (0.56–0.72)	0.65 (0.57–0.73)	0.63 (0.55–0.71)
Reader 2					
Photopenic or minimal to mild	36/49 (73)	129/141 (91)	1.0	1.0	1.0
Moderate or marked	13/49 (27)	12/141 (9)	4.1 (1.6–10.2)	4.0 (1.6–9.9)	4.3 (1.7–10.9)
*p* value			0.002	0.003	0.002
AUC			0.65 (0.57–0.73)	0.66 (0.58–0.74)	0.64 (0.56–0.72)

*BI-RADS* Breast Imaging-Reporting and Data System, *BMI* body mass index, *BPU* background parenchymal uptake, *HRT* hormone replacement therapy, NA not applicable

^a^Numbers in parentheses are percentages

^b^Numbers in parentheses are 95 % CIs

**Fig. 3:**
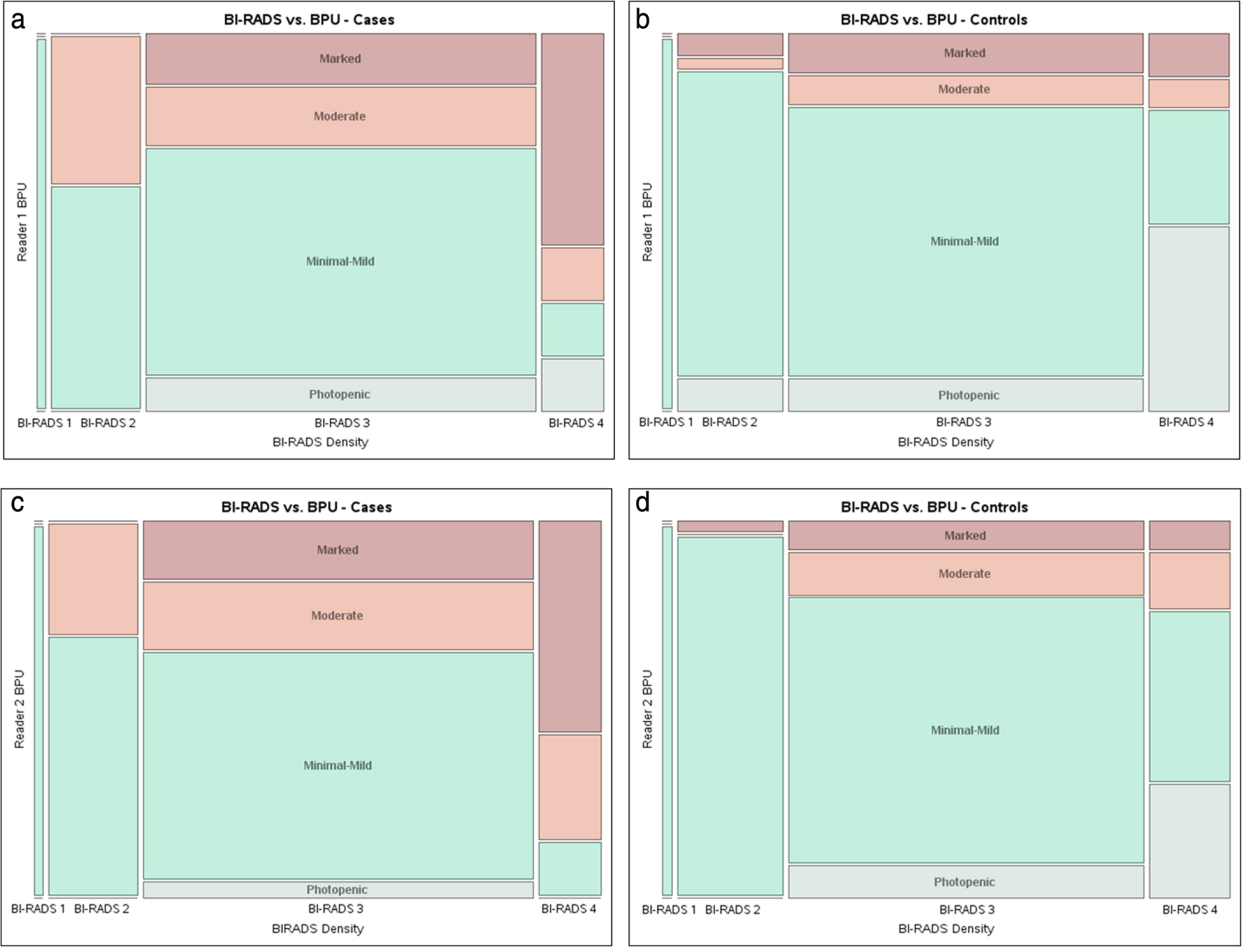
Mosaic plots show distribution of background parenchymal uptake (BPU) categories on molecular breast imaging (MBI) as a function of Breast Imaging-Reporting and Data System (BI-RADS) breast density categories for cases (**a**) and controls (**b**) interpreted by reader 1 and cases (**c**) and controls (**d**) interpreted by reader 2. BI-RADS 1 = almost entirely fat; BI-RADS 2 = scattered fibroglandular densities; BI-RADS 3 = heterogeneously dense; BI-RADS 4 = extremely dense

Neither BI-RADS density nor percentage density was statistically significantly associated with breast cancer in this study (BI-RADS ordinal OR 1.2, 95 % CI 0.7–2.0; percentage density OR [per 1 SD in square root of percentage density] 1.2, 95 % CI 0.8–1.8) (Table 1). As a result, adjustment for BI-RADS density (Table 3) and percentage density did not change BPU and breast cancer association (OR 3.3 [95 % CI 1.6–7.2] and OR 4.6 [95 % CI 2.1–10.5] for the two readers). BPU was associated with BI-RADS density in that the distribution of BPU categories differed across density categories overall (*p* < 0.0001), and among cases for one reader (reader 1, *p* = 0.04; reader 2, *p* = 0.10), and among controls for both readers (*p* < 0.001), as shown Fig. 3.

Additional adjustment for family history or Gail model 5-year risk within the model that included BMI and BI-RADS density adjustments had minimal impact on OR estimates. When additional adjustment for family history was made, the ORs for moderate or marked BPU vs. photopenic or minimal to mild BPU were 3.3 (95 % CI 1.5–7.2) and 4.7 (95 % CI 2.1–10.6), whereas additional adjustment for Gail model risk resulted in ORs of 3.5 (95 % CI 1.6–7.8) and 4.9 (95 % CI 2.1–11.5) for readers 1 and 2, respectively.

Moderate or marked BPU was more frequently observed among premenopausal women (26 [51 %] of 51 to 28 [55 %] of 51 for the two readers, respectively) than among postmenopausal women (25 [13 %] of 190 for both readers, *p* < 0.001). As shown in Table 3, the association of BPU with breast cancer remained in the postmenopausal subgroup for both readers. In the premenopausal subgroup, the association remained for reader 2 but did not reach statistical significance for reader 1. Among postmenopausal women, the results were unaffected with adjustment for use of hormone replacement therapy at the time of MBI. Finally, there was an elevated risk for the subset of cases and matched controls who had MBI with the reduced-dose protocol in June 2009 or later compared with those before June 2009 (OR 4.22 [95 % CI 1.55–11.52] and OR 8.08 [95 % CI 2.41–27.07] for the two readers), but there was no evidence for interaction (*p* = 0.28–0.45). The results were similar when we considered BPU assessment from the contralateral vs. ipsilateral breast for cancer cases and their respective matched controls (Additional files 2: Table S2).

The ability to discriminate between cases and controls, as assessed by the AUC, was 0.56 (95 % CI 0.48–0.63) for a model including BMI and BI-RADS mammographic density (age and menopausal status were matching variables). The addition of BPU to this model including BMI and density resulted in AUCs of 0.64–0.67 (Table 3).

## Discussion

We present the first evidence for BPU on MBI as a risk factor for breast cancer. The ORs of developing breast cancer for women with moderate or marked BPU, compared with those with photopenic or minimal to mild BPU, were 3.4 and 4.8 for two readers, respectively. This association was unaffected by adjustment for mammographic density measures. The association was also unaffected by use of postmenopausal therapy and remained in analysis limited to postmenopausal women only in order to eliminate potential cyclic effects on BPU seen in premenopausal women. The association between BPU and breast cancer also remained in analysis limited to invasive breast cancer cases only. Last, the association was similar in analyses performed using BPU assessed on the ipsilateral breast vs. contralateral breast, suggesting that BPU is a general marker of breast cancer risk rather than being specific to the side of the eventual cancer.

The magnitude of the associations of breast cancer risk with BPU found in this study are comparable to the association of breast cancer with biopsy findings of atypical hyperplasia (relative risk 4.2) [23], noting the possible relevance for BPU information to be used in risk stratification. As shown in this study and previous work, BPU can vary substantially among women with similar mammographic density [14], suggesting that BPU provides risk information beyond associations with density.

While mammographic density is established as an important breast cancer risk factor, a growing body of work supports the existence of additional anatomical and functional features of breast fibroglandular tissue that differ in discriminatory capacity for predicting breast cancer risk. For instance, extraction of breast density’s textural features has shown potential to improve risk discrimination over BI-RADS density categories and quantitative density assessment [24–26]. In contrast to these mammographic measures that depict fibroglandular tissue’s anatomical appearance, BPU on MBI provides a functional measure of Tc-99m sestamibi uptake, which is believed to be related to the presence of abundant mitochondria [27], cellular proliferation, and likely blood flow and angiogenesis as well [28].

Breast magnetic resonance (MR) imaging also depicts functional behavior of breast lesions and benign fibroglandular tissue through gadolinium-based contrast enhancement, known as *benign parenchymal enhancement* (BPE). Similar to our findings with BPU on MBI, BPE on MR imaging is influenced by hormonal and reproductive risk factors [29, 30] and has been associated with breast cancer diagnosis in two studies with case-control designs, reporting ORs of 3.3–10.1, though these studies were not restricted to incident breast cancer cases [31, 32]. Similarly to Tc-99m sestamibi, accumulation of gadolinium chelate agents in breast tumors is related to angiogenesis and vascular permeability [33]. One study has shown Tc-99m sestamibi uptake and gadolinium enhancement in background parenchyma to be correlated [34]. Several studies have shown a lack of correlation between BPE and mammographic density [35–37].

Within the Mayo Clinic practice, MBI is currently offered to the population of women with mammographically dense breasts (BI-RADS density c or d) who seek supplemental screening but do not meet criteria for breast MR (>20 % lifetime risk by familial risk models) [38]. Also, MBI is used for women in whom MR is recommended but cannot be performed due to contraindications, including inability or unwillingness to pay for an MR examination.

Supplemental MBI screening in dense breasts has been shown to offer a relatively high incremental cancer detection rate (8.8 cancers per 1000 women screened), a low false-positive rate (18 % for mammography with supplemental MBI), and lower cost per cancer detected compared with mammography alone [5, 39]. These factors, coupled with ability to now perform MBI at radiation doses acceptable for routine screening [40], support continued consideration of MBI as a suitable screening option for women with dense breasts. In addition to the role of MBI in supplemental screening, findings from this study also support further investigation into whether MBI may be useful as a risk prediction tool, especially if incorporated with other clinical risk predictors.

A strength of our study was the inclusion of only incident breast cancer cases, diagnosed at least 180 days after index MBI. This study design allowed us to establish BPU as a risk factor for future development of breast cancer, thus supporting the potential utility of BPU in risk prediction. While a case-control study including prevalent cases [31, 32] could identify factors associated with a current breast cancer diagnosis, it would not necessarily identify factors to be used in predicting subsequent risk. Further, by excluding cases at the time of index MBI as well as patients with breast cancer history who may have had noticeable treatment effects, we avoided introducing potential bias into the interpretation of BPU.

Our study had several limitations. First, BPU was assessed as a subjective measure, which led to some differences between the two readers. However, this subjective measure represents how MBI is currently read in practice, and the two readers agreed on BPU assessment in 94 % of subjects.

A second potential limitation of our study was that we did not have follow-up on approximately 6 % (183 of 3085) of patients in our MBI database from which the breast cancer cases were selected. Therefore, it is possible that we could have missed some breast cancers diagnosed outside our institution. However, we matched on known follow-up time, so our controls represented our cases and we do not anticipate a systematic bias.

Finally, the study data lacked specific information on day of menstrual cycle at which MBI was performed among premenopausal women. In a prior cross-sectional analysis including 417 premenopausal or perimenopausal women, no association between BPU category and follicular vs. luteal phase was found (*p* = 0.65) [14]. However, in a subsequent study of 42 premenopausal women with regular menstrual cycles, serial MBIs were timed to be performed at peak follicular and luteal phases. Within-woman analyses showed high BPU (moderate or marked) to be observed more frequently at luteal phase than follicular phase [15]. It is currently unclear whether menstrual cycle effects on BPU have associations with breast cancer risk. However, our finding of a strong association between breast cancer and BPU in the postmenopausal subgroup, even after adjustment for hormone therapy, suggests that the association of BPU with breast cancer is not simply an artifact of premenopausal cyclic variation in BPU.

Our study included primarily women with mammographically dense breasts (80 % were either heterogeneously or extremely dense). The low number of women with nondense breasts likely accounts for the lack of a statistically significant association between mammographic density measures and breast cancer observed in this population. However, since this population is the target subgroup that would receive MBI, it is appropriate for our investigation.

## Conclusions

BPU, which describes the functional uptake observed within fibroglandular tissue on MBI, is strongly associated with breast cancer risk, and this association remained after adjustments for mammographic density or postmenopausal hormone use. For women with dense breasts, who comprise over 40 % of the screening-eligible population, BPU may serve as an additional risk factor to help identify the subgroup most likely to benefit from tailored screening or primary prevention options.

## Additional files

Additional file 1: Table S1. Characteristics of 62 incident breast cancer cases. (DOC 51 kb) Additional file 2: Table S2. Association of background parenchymal uptake (BPU) with breast cancer for ipsilateral vs. contralateral side. (DOC 43 kb)

## Abbreviations

ACR: American College of Radiology
BCSC: Breast Cancer Surveillance Consortium
BI-RADS: Breast Imaging-Reporting and Data System
BMI: body mass index
BPE: background parenchymal enhancement
BPU: background parenchymal uptake
DCIS: ductal carcinoma in situ
HRT: hormone replacement therapy
IQR: interquartile range
LCIS: lobular carcinoma in situ
MBI: molecular breast imaging
MR: magnetic resonance
NA: not applicable
