# Supplementary material for: Background parenchymal uptake on molecular breast imaging as a breast cancer risk factor: a case-control study
Source: Breast Cancer Res. 2016 Apr 26;18:42. doi: 10.1186/s13058-016-0704-6 (PMC4845425; doi:10.1186/s13058-016-0704-6)
Supplement: Additional file 1: Table S1. — Characteristics of 62 incident breast cancer cases. (DOC 51 kb) [file 13058_2016_704_MOESM1_ESM.doc]

Table S1. Characteristics of 62 incident breast cancer cases.

| **Characteristic** | **No./total (%)** |
| --- | --- |
| **Years from index MBI to diagnosis (y)*** | 3.3 ± 2.0 (0.5 – 8.8) |
| **Mode of detection** |  |
| Screen mammography | 47/62 (76) |
| Screening MBI | 2/62 (3) |
| Screening MRI | 6/62 (10) |
| Interval cancer, patient-reported symptoms | 7/62 (11) |
| **Tumor size (cm)*** | 2.0 ± 2.3 (0.1 – 13.0) |
| **Invasive cancers (N = 45)** |  |
| Histology |  |
| Ductal | 36/45 (80) |
| Lobular | 4/45 (9) |
| Mixed | 5/45 (11) |
| Histologic grade |  |
| I | 15/45 (33) |
| II | 21/45 (47) |
| III | 8/45 (18) |
| Unknown^†^ | 1/45 (2) |
| Tumor size* | 1.7 ± 2.1 (0.1 – 13.0) |
| Lymph node involvement |  |
| Positive | 6/45 (13) |
| Negative | 39/45 (87) |
| Hormone receptor status |  |
| ER positive and PR positive | 36/45 (80) |
| ER positive and PR negative | 6/45 (13) |
| ER negative and PR negative^‡^ | 3/45 (7) |
| HER2 status |  |
| Positive | 8/45 (18) |
| Negative^‡^ | 37/45 (82) |
| **DCIS (N = 17)** |  |
| Histologic grade |  |
| Low | 2/17 (12) |
| Intermediate | 6/17 (35) |
| High | 9/17 (53) |
| Tumor size* | 2.8 ± 2.8 (0.2 – 9.0) |
| Hormone receptor status |  |
| ER positive and PR positive | 12/17 (80) |
| ER positive and PR negative | 3/17 (18) |
| ER negative and PR negative | 2/17 (12) |

Note – Unless otherwise noted, data are number of patients and data in parentheses are percentages. DCIS = ductal carcinoma in situ. ER = estrogen receptor. PR = progesterone receptor. HER2 = human epidermal growth factor receptor 2.

*Data are mean ± standard deviation. Data in parentheses are the range.

^†^One breast cancer case with unknown grade of invasive cancer was a microinvasion among high grade DCIS.

^‡^Two women were diagnosed with triple negative (ER, PR, and HER2 negative) breast cancer.
