# Supplementary material for: Background parenchymal uptake on molecular breast imaging as a breast cancer risk factor: a case-control study
Source: Breast Cancer Res. 2016 Apr 26;18:42. doi: 10.1186/s13058-016-0704-6 (PMC4845425; doi:10.1186/s13058-016-0704-6)
Supplement: Additional file 2: Table S2. — Association of background parenchymal uptake (BPU) with breast cancer for ipsilateral vs. contralateral side. (DOC 43 kb) [file 13058_2016_704_MOESM2_ESM.doc]

Table S2. Association of background parenchymal uptake (BPU) with breast cancer for Ipsilateral vs. Contralateral side.

| **BPU** | **Breast Cancer Cases*** | **Controls*** | **Odds Ratio,**  **adjusted for BMI**^†^ |
| --- | --- | --- | --- |
| **BPU determined by ipsilateral breast** |  |  |  |
| **Reader 1** |  |  |  |
| Photopenic or Minimal-mild | 42/62 (68) | 152/179 (85) | 1.0 |
| Moderate or Marked | 20/62 (32) | 27/179 (15) | 3.2 (1.5, 7.2) |
| P-value |  |  | 0.004 |
| AUC |  |  | 0.62 (0.55, 0.69) |
| **Reader 2** |  |  |  |
| Photopenic or Minimal-mild | 41/62 (66) | 152/179 (85) | 1.0 |
| Moderate or Marked | 21/62 (34) | 27/179 (15) | 3.6 (1.6, 7.7) |
| P-value |  |  | 0.001 |
| AUC |  |  | 0.61 (0.54, 0.68) |
| **BPU determined by contralateral breast** |  |  |  |
| **Reader 1** |  |  |  |
| Photopenic or Minimal-mild | 41/62 (66) | 150/179 (84) | 1.0 |
| Moderate or Marked | 21/62 (34) | 29/179 (16) | 3.1 (1.5, 6.5) |
| P-value |  |  | 0.003 |
| AUC |  |  | 0.63 (0.56, 0.70) |
| **Reader 2** |  |  |  |
| Photopenic or Minimal-mild | 41/62 (66) | 156/179 (87) | 1.0 |
| Moderate or Marked | 21/62 (34) | 23/179 (13) | 4.8 (2.1, 10.9) |
| P-value |  |  | 0.002 |
| AUC |  |  | 0.65 (0.58, 0.72) |

*Numbers in parentheses are percentages.

^†^Numbers in parentheses are 95% confidence intervals.

BMI = body mass index
